# Self-Assembling Peptide Hydrogels as Functional Tools to Tackle Intervertebral Disc Degeneration

**DOI:** 10.3390/gels8040211

**Published:** 2022-03-31

**Authors:** Cosimo Ligorio, Judith A. Hoyland, Alberto Saiani

**Affiliations:** 1 Department of Materials, School of Natural Sciences, Faculty of Science and Engineering, The University of Manchester, Manchester M1 3BB, UK; a.saiani@manchester.ac.uk; 2 Manchester Institute of Biotechnology (MIB), The University of Manchester, Manchester M1 7DN, UK; 3 Division of Cell Matrix Biology and Regenerative Medicine, School of Biological Sciences, Faculty of Biology, Medicine and Health, The University of Manchester, Manchester M13 9PG, UK; judith.a.hoyland@manchester.ac.uk

**Keywords:** self-assembling peptide hydrogels, intervertebral disc, tissue engineering

## Abstract

Low back pain (LBP), caused by intervertebral disc (IVD) degeneration, is a major contributor to global disability. In its healthy state, the IVD is a tough and well-hydrated tissue, able to act as a shock absorber along the spine. During degeneration, the IVD is hit by a cell-driven cascade of events, which progressively lead to extracellular matrix (ECM) degradation, chronic inflammation, and pain. Current treatments are divided into palliative care (early stage degeneration) and surgical interventions (late-stage degeneration), which are invasive and poorly efficient in the long term. To overcome these limitations, alternative tissue engineering and regenerative medicine strategies, in which soft biomaterials are used as injectable carriers of cells and/or biomolecules to be delivered to the injury site and restore tissue function, are currently being explored. Self-assembling peptide hydrogels (SAPHs) represent a promising class of *de novo* synthetic biomaterials able to merge the strengths of both natural and synthetic hydrogels for biomedical applications. Inherent features, such as shear-thinning behaviour, high biocompatibility, ECM biomimicry, and tuneable physiochemical properties make these hydrogels appropriate and functional tools to tackle IVD degeneration. This review will describe the pathogenesis of IVD degeneration, list biomaterials requirements to attempt IVD repair, and focus on current peptide hydrogel materials exploited for this purpose.

## 1. Introduction

### 1.1. Structure and Function of the IVD

**Figure 1:**
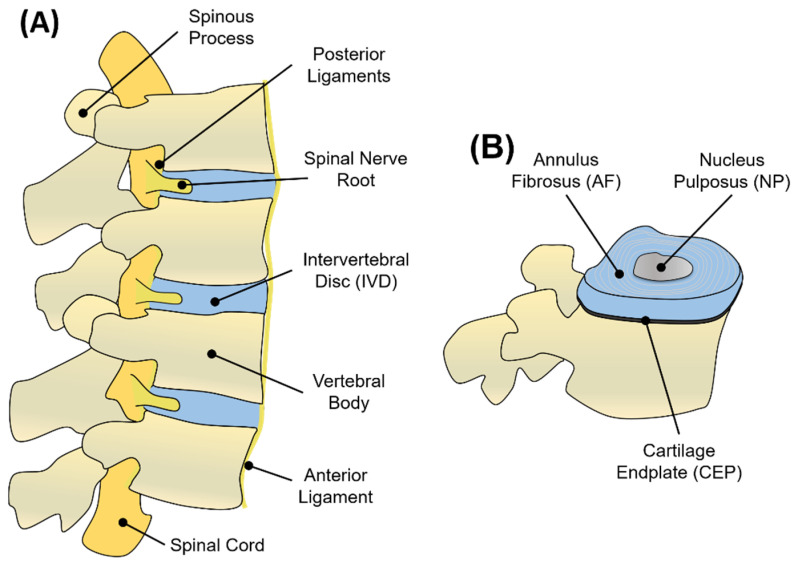
(**A**) Graphical representation of adjacent vertebral units in sagittal view. Each unit consists of vertebral bodies surrounding an IVD. The nerve supply of the IVD and vertebral bodies consists of the spinal cord disposed longitudinally along the vertebrae and passing through the intervertebral foramen. Vertebrae are kept in place by anterior and posterior ligaments. (**B**) Schematic representation of the IVD, showing the NP, AF, and CEP regions.

The IVD, the building block of the spinal motion segment, was anatomically described for the first time at the University of Padua by the famous Italian anatomist Andreas Vesalius in 1555, who introduced the intervertebral body to early anatomists [1]. The human spine is composed of a total of at least 23 IVDs interposed between adjacent vertebrae, joining them from the axis to the sacrum [2]. The IVDs constitute one-third of the total spinal column’s length, with 6 IVDs present in the cervical region, 12 in the thoracic region, 5 in the lumbar region, and 1 between the sacrum and the coccyx [2]. Each IVD is a pad of fibrocartilage, 7–10 mm thick and with an average diameter of 4 cm, which is composed of three integrated tissues: the nucleus pulposus (NP), the annulus fibrosus (AF), and the cartilage endplates (CEP) [3] (Figure 1). Schematically, the AF represents a thick outer ring of fibrous cartilage comprised of concentric layers of type I collagen that circumferentially surrounds a proteoglycan (PG)-rich gelatinous core (the NP tissue) to constitute a composite tissue sandwiched inferiorly and superiorly by two thin layers of cartilage, better known as CEP [3]. Fibres from the inner AF tissue bridge this tissue to CEP, while fibres coming from the outer layer of AF are anchored within the bony endplates of vertebrae to form a continuous vertebral body. Longitudinal ligaments (anterior and posterior) laying at the front and at the back of the vertebral body, along with the spinous processes, constitute a ‘three-joint complex’ called the vertebral unit (Figure 1). As the name suggests, the vertebral unit represents the unit of the column, dictating its biomechanics and biological function [2].

Biomechanically, the IVD acts as a shock absorber by transmitting loads along the spine. External loads exerted on the spine are mainly related to body weight and muscle traction to maintain equilibrium, but also different postures, body motion, and carrying weights are referred to as stress components acting on the column [4]. It has been estimated that IVDs are able to tolerate multiple cycles of loading such as compression, tension, torsion, and bending up to 2.5 MPa, from a minimum of 0.1 MPa for simple resting to a maximum of 2.3 MPa for carrying a weight of 20 kg in a flexed position [5]. The biomechanics of the IVD can be approximated as a mechanical interplay existing between the NP and AF. Under compression, hydrostatic pressure is created within the gelatinous NP, which in turn generates tensile stress (or ‘hoop’ stress) in the surrounding cartilaginous layers of the AF to counteract the original load [6]. The success behind this biomechanical response relies on the NP’s high water content, which is needed to generate the hydrostatic pressure that increases in response to compressive loading, along with a high shear resistance and complex collagen network between AF’s lamellae, which enable the AF to develop tensile stress opposite to the NP’s pressure [6]. This biomechanical balance is essential to absorb mechanical forces and dissipate their energy. Moreover, under compression, the limited swelling of the CEP and its elasticity are fundamental to reducing the rate of water expelled from the pressurised NP, allowing the NP to swell and expand [6].

### 1.2. Nucleus Pulposus (NP)

**Figure 2:**
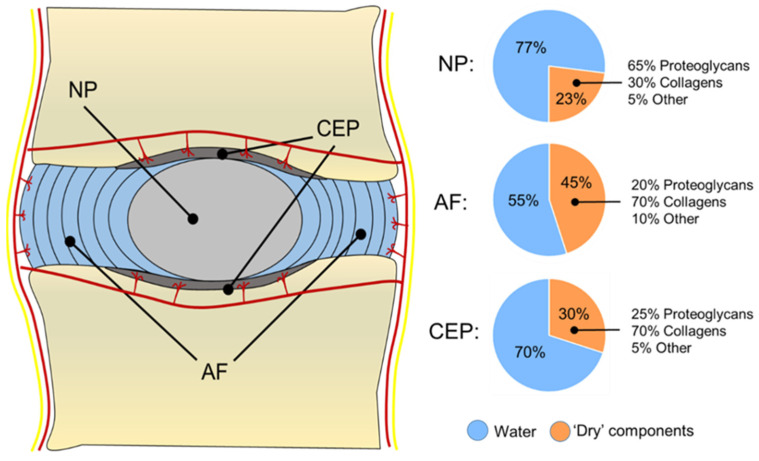
Schematic representation of the human IVD, showing its water and ECM content. The IVD is surrounded by blood and nerve vessels. Capillaries penetrate a few millimetres into the outer AF to provide nutrients and waste exchange. Cells from the avascular NP and inner AF receive nutrients and are able to exchange waste products through a bidirectional flow occurring via blood capillaries that penetrate the subchondral plate and reach the CEP.

The NP is a soft, highly hydrated and gelatinous tissue that occupies the central region of the IVD (Figure 1B). This tissue appears translucent and gel-like in the healthy state, with a heterogeneous structure composed of water, glycosaminoglycans (GAGs), collagen fibres, mineral salts, and cellular components [7]. The amount of water in the NP is around 80–90% in the early life, with a progressive decrease up to 70% at ages over 60, when the NP becomes white in colour, fibrotic, and amorphous [2,7]. Structurally, the NP can be considered as a three-dimensional (3D) viscoelastic network mainly composed of a PG-rich gel, in which multiple populations of collagen and non-collagenous proteins are dispersed. PGs represent up to 65% of the NP’s dry weight in the early stages, and they are responsible for binding water within the tissue due to their negatively charged residues [8]. Although many comparisons are made between the NP and the articular cartilage (AC), in the NP, the proteoglycans (PGs) to fibrillar collagen ratio has been reported to be 27:1 compared to the 2:1 ratio for AC, making the NP a more hydrated and softer tissue compared to AC [9]. Aggrecan represents the most abundant PG in the NP, although traces of other GAGs, including hyaluronan, chrondroitin sulphates, decorin, lumican, biglycan, fibromodulin, and versican, have also been reported in the NP tissue [10,11,12]. In this PG-rich network, collagen fibres (principally collagen type II) are randomly dispersed and highly interconnected with PG fibres, constituting 15–20% of the dry weight (Figure 2). Collagen fibres, including type II, VI, IX, and XI, form a loose, secondary interpenetrating network, which has the scope with PGs to hold the NP together [13,14]. The remaining dry weight, accounting for 10–25%, is represented by non-collagenous fibres (mainly elastin up to 150 μm in length), which are arranged radially across the NP [12].

In this ECM, the cell population of the NP is derived from the notochord during embryonic development, with large vacuolated embryonic notochordal cells (25–85 μm in size) that are gradually replaced by smaller and rounded ‘chondrocyte-like’ NP cells (10 μm in size) in juvenile discs, which have a lower metabolic activity than notochordal cells [15]. NP cells are sparse within the tissue, with an average cell density (4000 cells mm^−3^) lower than the AF and CEP, as well as three-fold lower than the average number in AC [16,17]. Although NP cells have a similar morphology to AC chondrocytes, an extensive gene expression profiling conducted by Minogue et al. on human NP cells has identified a pool of specific markers upregulated in NP cells but not articular chondrocytes. These studies have allowed an improved characterisation of NP cells, making the comparison between the two cell types clearer [18,19]. The number of NP cells within the tissue tends to decrease with age, when an increased number of macrophages, T-cells, and lymphocytes start to populate the tissue due to blood vessel infiltration, contributing to the pro-inflammatory milieu that affects NP tissue during fibrosis degeneration and herniation [20,21]. Moreover, the normal NP is an avascular and aneural tissue, where NP cells are far at least 8 mm from the nearest blood supply [22], which makes nutrients and waste exchange based solely on diffusion and self-healing extremely difficult to achieve [23]. In this scenario, NP cells rely on anaerobic cellular respiration to produce adenosine triphosphate (ATP) for their vital functions, so lactate as the by-product of glycolysis is largely accumulated in the NP. This makes the NP a relatively hostile microenvironment, with a relatively low pH (pH < 6.5) and low oxygen content (O_2_ levels < 5%) [24].

### 1.3. Annulus Fibrosus (AF)

The NP is circumferentially surrounded by the AF, which is composed of 15–25 concentric rings made of alternating lamellae of collagen type I inclined by 60° with respect to the main axis of the column [25] (Figure 1B). Although a clear demarcation region between NP and AF was drawn historically [2], it has been proved that the two tissues are extremely integrated, with a thin region between the two called the transition zone (TZ) having increased metabolic activity and sensitivity to normal forces and growth factor (GF) modulation [26]. This region is fundamental for the NP to expand at the expense of the AF, while for the AF, this region is necessary to sense the hydrostatic pressure coming from the NP and exert a mechanical resistance to counteract it [26]. The building blocks of the AF are the lamellae, which are 50–500 μm-thick collagen layers with thickness increasing from the inside to the outside [14,25]. Each lamella is composed of 100–200 nm collagen type I fibre bundles, uniformly oriented at 60° to the vertical axis, alternating to the left and right between adjacent lamellae [14,25]. The interconnection between lamellae is little, and elastin fibres have been found among collagen fibrils within the same lamellae [12]. This matrix organisation makes the lamellae prone to expand during compression, allowing different degrees of movement, still ensuring elastic recoil to its original position when the stress (e.g., bending, tension, and compression) is suppressed. The AF can be divided into the inner and outer annulus according to the distance to the NP. Moving from the inner part to the outer, the content of PGs, water, and collagen type II decreases, while the content of collagen type I increases, making the outer rings of AF stiffer and elastic, while the inner rings are softer and more deformable. As also occurs for tendons, collagen type I provides tension resistance in the AF, while collagen type II fibres intertwined with PGs help to bind and keep water molecules together in order to keep the AF stable against large compressive forces [13,27]. In the AF, all the collagen types represent 50–70% of the total dry weight (with collagen I being the dominant type), while 20–25% is provided by PGs and the remaining 5–10% by elastin fibrils [7] (Figure 2).

Regarding the cellular component, the AF is populated by elongated, fibroblast-like cells that tend to align to the collagen fibres within the lamellae, especially in the outer rings of the tissue [2]. The cells of the AF present large protrusions (up to 30 μm long) that have been not observed in AC, which have been suggested to act as mechanical strain sensors within the tissue to regulate shock absorbance [28]. Like the NP, the AF is largely avascular and aneural, although evidence of penetrating blood vessels and sensory nerve fibres up to a few millimetres in the outer rings have been found, which may contribute to nutrient transport and waste removal [23]. However, substantial nerve and blood vessel infiltration occur during IVD degeneration, which, coupled with a limited self-healing capability, makes AF injuries difficult to repair without surgical intervention [29,30].

### 1.4. Cartilage Endplate (CEP)

The third component of the IVD is represented by the CEP (Figure 1B). These endplates are thin layers of hyaline cartilage (~0.6 mm) that interface the avascular AF and NP to the highly vascularised vertebrae, specifically with vertebral bodies’ endplates called bony endplates [31]. The thickness of the CEP is thinner at the centre and larger at the periphery so that NP tissue that stands at the core of the IVD can swell and expand easily by pushing on the CEP’s inner surface [32]. The CEP is fundamental to maintaining the IVD and the vertebral compartments as distinct mechanically entities, but at the same time, it allows them to communicate biochemically by ensuring nutrition and waste exchange through capillaries and blood vessels that pass through the endplates into the IVD. Interestingly, Nachemson et al. showed that only the central part of the CEP is permeable to the diffusion of dyes or radioactive substances, leaving the rest of the nutrients mainly supplied by a network of vascular channels within the vertebral bodies [33].

Being constituted of hyaline cartilage, the CEP’s dry weight is mainly composed of collagen fibres (75% of the dry weight), e.g., collagen type II, but also type III, VI, and X, as well as PGs such as decorin and biglycans (25% of the dry weight, Figure 2). The collagen content is higher at the periphery, while the PG content is higher at the centre of the CEP to allow mechanical compliance [34,35]. Collagen fibres within the CEP run horizontally to its surface in contact with the IVD, but some fibres also continue to the disc merging with fibres coming from the AF, as well as anchoring with the bone trabeculae of the bone endplates to ensure connections along the column [31]. The CEP is partly mineralised, but it is also populated by a family of chondrocytes that can become hypertrophic, especially during IVD degeneration, when CEP’s calcification is increased, and the flow of nutrients and waste exchange is highly affected [36].

### 1.5. Anabolic Growth Factors Present in the IVD

As defined by Hynes, native ECMs are not ‘just pretty fibrils’, but they are a reservoir of growth factors (GFs), which are bound to the ECM proteins or dispersed in the ECM as soluble factors [37]. Indeed, every tissue for its differentiation and maturation require specific GFs, which are the master regulators of intracellular pathways. A broad range of GFs is known to be present in the IVD, including many members of the transforming growth factor β (TGF-β) superfamily. In particular, TGF-β molecules play an important role in IVD growth and homeostasis. TGF-β signalling orchestrates tissue formation at the embryonic stage via notochord condensation and supports tissue homeostasis at postnatal stages by promoting the synthesis of PGs and collagens, which dictate the correct structure and functionality of the native NP [38]. Gruber et al., for example, demonstrated that TGF-β1 could induce cell proliferation and reduce disc cell apoptosis, as well as promote ECM synthesis in vivo [39]. Similarly, Jin and co-workers observed that the suppression of TGF-β3 signalling in mice causes a notable reduction in the area and length of CEP tissue, resulting in NP cells being deprived of nutrients and prone to adopt a catabolic, pro-inflammatory phenotype [40]. Along with TGF-β molecules, growth differentiation factor (GDF) molecules are also highly involved in tissue homeostasis and matrix turnover in the IVD. Recombinant GDF-5, for example, has shown increased PGs and collagen type II gene and protein expression *in vitro*, both with mouse and bovine NP cells [41]. Similarly, GDF-6 has been shown to play a crucial role in the development of bones and joints by forming morphogenetic gradients during development, while knockout of GDF-6 in the disc reveals spine defects and reduced PG content in mice [41]. Due to their biological function, GFs, along with ECM components, should be considered active players and regulators of IVD during health and disease.

## 2. Pathophysiology of Intervertebral Disc Degeneration

### 2.1. Alterations in the ECM of Degenerated Discs

In the IVD, resident cells are responsible for maintaining a tight balance between anabolic and catabolic processes so that any imbalance towards degradative processes can lead to matrix catabolism and tissue breakdown in the IVD. Although the exact causes of this imbalance are not fully elucidated, new evidence has highlighted several factors that may contribute to, or may be responsible for, IVD degeneration. ECM breakdown, altered matrix synthesis, and changes in cell number, phenotype, and behaviour are characteristic features of the IVD [42]. During the early stages of degeneration, disc cells adopt a defensive mechanism by increasing their secretion of collagen type II molecules in an attempt to counteract ECM degradation and start a repair mechanism [43]. This phenomenon is only temporary since, with advancing degeneration, collagen type II synthesis shifts dramatically to collagen type I, specifically in the NP and in the inner AF [7,44]. In the NP, the collagen fibres shift more to the type I form and become less packed and looser due to a decrease in the pyridinoline cross-links between collagen fibres. This conformational change affects the biomechanics of the NP, which starts to become stiffer and granular [43]. A decrease in collagen type II synthesis is also accompanied by an increase in the production of collagen type X, which causes NP’s hypertrophy and CEP calcification [45]. In particular, calcified CEP results in a limited diffusion of nutrients and waste into and out of the NP so that lactate accumulates, leading to tissue acidification. In healthy IVD, the pH has been shown to be ~7.1, while during degeneration, it can drop to 6.8 (mildly degenerated discs) or 6.5 (severely degenerated discs) [46]. Low pH values have been associated with reduced cell viability, as well as the reduced production of collagen and PGs in several 2D and 3D studies [47]. Gilbert and co-workers, for example, showed that human NP cells exposed to acidic pH (6.8 and 6.5) are able to sense low pH via acid-sensing ion channel-3 proteins (ASICs) proteins, resulting in a catabolic and degenerated phenotype for NP cells. Interestingly, when ASICs were inhibited, proinflammatory and pain-related makers in NP cells were downregulated, suggesting a correlation between low pH and catabolic phenotype [48].

**Figure 3:**
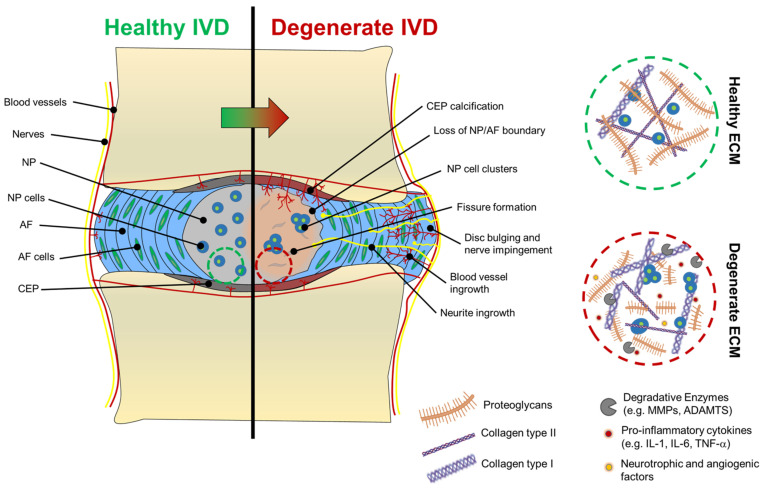
Macroscopic and microscopic changes of the IVD during degeneration. Characteristic changes of the NP’s ECM after degeneration are illustrated in the dashed circles. ECM of degenerated discs (dashed red circle) shows shorter aggrecan macromolecules and more collagen type I fibres (thicker fibre bundles) than collagen type II (thinner collagen bundles), which are largely abundant in the ECM of healthy discs (dashed green circle).

In addition to changes in collagen expression, the disc also experiences a decrease in PGs [49]. Indeed, although degenerated NP cells are still capable of producing aggrecan and versican, these molecules become shorter and fragmented so that the overall content of PGs dramatically decreases [50] (Figure 3). As the aggrecan molecules of degenerated discs are disrupted and are in the form of fragments, they can only retain smaller quantities of water than their fully formed version, and they can easily leach out of the tissue due to their smaller dimensions [3]. During degeneration, as the content of aggrecan decreases, the synthesis of versican, biglycan, and decorin increases [44]. However, versican contains fewer chondroitin sulphate chains than aggrecan, so they cannot compensate for the water binding and high osmotic pressure lost with aggrecan degradation [3]. The result is a tissue that is less able to retain water and becomes dehydrated over time. Moreover, increased production of collagen type I leads the NP to become more fibrous and less compliant as the degeneration progresses. Among the other ECM molecules, fibronectin is another ECM component whose content as a fragmented molecule increases during disc degeneration [51]. It has been demonstrated, in fact, that fragments of fibronectin (~30 kDa) can induce catabolic cytokine production and stop aggrecan synthesis in vitro [52], while *in vivo*, they have been associated with the loss of PGs and osteophyte formation [53].

### 2.2. ECM Degradation in Degenerated Discs

A range of degradative enzymes and catabolic cytokines are released during disc degeneration. An increased expression of matrix metalloproteinases (MMPs) and ‘a disintegrin and metalloproteinase with thrombospondin motifs’ (ADAMTS), which are involved in the cleavage of the major components of the ECM, have been reported during degeneration. MMP-1, MMP-8, and MMP-13, for example, are able to degrade collagen type I and II, while MMP-2 and MMP-9 have been more generally associated with the degradation of triple helices along with gelatinases [54]. MMPs are also able to degrade aggrecan molecules at specific sites (G1–G2 and G2–G3 interglobular domains), although their activity is less pronounced than ADAMTS [55]. An impairment between ADAMTS and the tissue inhibitors of MMPs (TIMPs) can lead to matrix degradation, in particular through aggrecan fragmentation, which is a feature of degenerated discs [56].

It has been hypothesised that an increased expression of ADAMTS, especially ADAMTS-1, ADAMTS-4, and ADAMTS-9 in degenerated discs, could be potentially regulated by a series of proinflammatory cytokines and inflammatory mediators, such as interleukins (ILs), tumour necrosis factor-α (TNF-α), and nitric oxide, which are released by NP cells in the cellular microenvironment during degeneration [57]. These soluble regulators of cellular function are involved together, with some of them acting as master regulators of others. IL-6, for example, is thought to play indirectly by inducing NP cells to respond to IL-1 and TNF-α [58]. IL-1, in its two isoforms (IL-1α and IL-1β), along with its receptors (IL-1RI and IL-1RII) and their natural receptor antagonist (IL-1Ra), have all been identified in the IVD. In particular, IL-1 and IL-1Ra are in equilibrium during homeostasis, while an increase in the former compared to the latter has been observed in degenerated discs [59]. Strong evidence suggests that increased production of IL-1β by NP cells represents a master regulator of disc degeneration. Indeed, Le Maitre et al. showed that the administration of recombinant IL-1 to NP cells in vitro induced an increased production of MMP-3, MMP-13, and ADAMTS- 4, along with a collagen II-to-collagen I shift and a decrease in aggrecan production, which are all signs of matrix breakdown and progressive degeneration [60]. On the other hand, IL-1Ra directly delivered into explants of degenerated IVDs has been shown to halt matrix degradation and start to reverse the typical signs of degeneration, highlighting the importance of the IL-1/IL-1Ra balance to keep the NP in its healthy state [61,62].

### 2.3. Nerve and Blood Vessel Infiltration in Degenerated Discs

One of the aspects that makes disc degeneration painful and debilitating is associated with vascular and nerve ingrowth during degeneration. At birth, only AF and CEP possess vascular networks, which recede with age so that only the endplates are vascularised, while NP and AF remain avascular and aneural [2]. During disc degradation, a decreased aggrecan content has been associated with the increased severity of degradation and neovascularisation, suggesting that blood vessels infiltration is avoided in a healthy state by aggrecan molecules themselves, which are thought to inhibit endothelial cell adhesion and migration [63]. Moreover, neovascularisation and innervation are also associated with the formation of fissures within the AF and NP, suggesting that tissue breakdown allows blood and nerve vessels to interdigitate within these tissues. In the last decade, biochemical links between neovascularisation and inflammation have been identified. An increase in IL-1β has been associated with the stimulation of angiogenic and neurogenic factors, such as vascular endothelial GF (VEGF), nerve GF (NGF), and brain-derived neurotrophic factor (BDNF) [42]. Both NP and AF, in their healthy state, have low levels of NGF and BDNF, but their levels increase notably during degeneration [30]. To support this, Purmessur et al. found that levels of NGF and BDNF can be increased by the addition of IL-1β and TNF-α to cultured human NP cells and that substance P, a molecule involved in pain sensation or nociception, can be highly expressed [30]. Driven by neurotrophic factors, neurites start to infiltrate NP and the AF tissues. In degenerated discs, nerve vessels are found alongside blood vessels, probably formed because of infiltrating endothelial cells that, during angiogenesis, secrete NGF [42]. Neurites found in the NP show a similar expression profile to neurites found in the AF, suggesting that neoinnervation may have a common source. The presence of NGF and BDNF in the cell microenvironment can also activate the NF-kB pathway, which is responsible for the secretion of further pro-inflammatory cytokines and degradative enzymes that accelerate disc degradation [64,65]. In addition to the pain caused by neurite infiltration (discogenic pain), degenerated discs often collapse and bulge out of the vertebral unit, pushing on the spinal nerves exiting the IVD foramen, causing nerve compression and additional pain [66]. Macroscopic and molecular features of IVD degeneration are shown in Figure 3.

### 2.4. The Effect of Ageing on Disc Degeneration

During development, NP tissue is populated by large vacuolated cells known as notochordal cells because of their origin, which are metabolically active and produce large amounts of PGs [67]. By the age of 10, notochordal cells are replaced by smaller chondrocyte-like cells known as NP cells, which are less metabolically active. The period within which notochordal cells start to disappear (3–10 years old) is accompanied by a high level of cell death [67]. In this period, the NP tissues lose their characteristic gel-like structure of the early years and become less hydrated, cartilaginous, and fibrous. These years also coincide with the earliest identifiable signs of disc degeneration ever reported through magnetic resonance imaging (MRI) [3]. It is clear that the passage from a cell population predominantly of notochordal cells to a population of NP cells and the changes in matrix composition and mechanics are the earliest signs of disc changes occurring with ageing. Cell death, occurring through apoptosis [68] and autophagy [69,70], comes in cycles followed by proliferation. Between 11 and 16 years, NP cells undergo a notable cell proliferation, which is thought to follow the massive cell death that occurs between ages 3 and 10 [67].

Cells during proliferation tend to create clusters, which, in turn, predispose the NP cells to replicate senescence traits [71]. In the last decade, several markers of cell senescence have been identified in degenerated discs [68,72,73]. Le Maitre and co-workers, for example, found that during degeneration, there is an increase in the protein and gene levels of p16^INK4A^, which is a cell cycle inhibitor, accompanied by a decrease in the average telomere length (characteristic of ageing) and an increase in senescence-associated β-gal staining [72]. Similarly, Kim et al. a few years later, reported that the telomere-based p53-p21-pRB pathway plays a crucial role in premature senescence in NP cells, confirming that with degeneration, senescent cells accumulate in the disc [73]. Senescence has been shown to also be associated with an increase in matrix catabolism since elevated gene expression of MMPs and ADAMTS were recorded in aged discs [74]. Although these studies and more suggest that there is a link between senescence, ageing, and disc degeneration, it is still not clear if the link is causative, i.e., if cell senescence causes IVD degeneration. However, evidence suggests that degeneration is accompanied by cell senescence, autophagy, and apoptosis, which are features of tissue ageing [75].

### 2.5. Mechanical Factors for Disc Degeneration

Although it has been reported that intense exercise has no adverse effect on the disc, experimental overloading and injury to the disc can be considered inductive factors for disc degeneration. In a study by Puustjärvi et al., for example, beagles were used as animal models to study the changes in PGs in two IVDs (cervical C5 and thoracic T6) after one year of running exercise on a treadmill (40 km per day) compared to non-running control dogs [76]. The animals subjected to extensive exercise showed an increase in PGs in the C5 disc and a reduction in T6 discs, in particular within the AF tissue. Although PG content is related to disc hydration and viscoelasticity, this study highlighted that PG content might be affected by a spine-location dependency after exercise, which can alter the biomechanics of the entire spinal column [76]. In another animal model, Iatridis et al. applied external compressive forces to the tails of rats to recreate spine overloading [77]. Chronically applied compressive forces resulted in a decreased disc height and loss of spine flexibility. The same effects were observed with rats’ tail immobilisation, but earlier and with a larger magnitude. Similarly to Puustjärvi’s work, the content of PGs in the overloading-induced group was increased, but the original biomechanical features of the disc, such as flexibility and angular laxity, were lost [77].

Along with macroscopic effects on spine mobility, mechanical loadings can also have a profound effect on the ECM organisation and phenotype of disc cells, which are ultimately the functional units of the IVD. Different in vitro studies have demonstrated that the magnitude, frequency, and duration of loading play a pivotal role in determining cell response and inducing consequent degeneration [78,79,80]. In two separate studies, Handa first [81] and Neidlinger-Wilke et al. later [82] showed that low-magnitude loadings could increase PG and collagen synthesis (aggrecan and collagen type II) in human disc cells and human nucleus explants, while the increased production of matrix degradative enzymes (mainly metalloproteinases-3, MMP-3) was reported with high-magnitude loadings. Once disc cells initiated a catabolic response and were degenerated, their mechanical response was largely affected, probably due to an alteration at the mechanotransduction level. Le Maitre and co-workers, for example, encapsulated AF and NP cells in alginate beads and applied dynamic hydrostatic pressure to the hydrogels (>2 MPa) to mimic the pressure experienced by cells *in vivo*. In non-degenerated cells, typical markers of disc matrix production such as SRY-box transcription factor-9 (SOX-9), collagen type II, and aggrecan were upregulated, with no effect on degradative enzymes [83]. On the contrary, degenerated cells showed no response to mechanical load, with no effect on the same gene target [83]. Similarly, Gilbert et al. showed that cyclic tensile strain applied at 1 Hz could reduce the anabolic response of AF cells derived from degenerated discs, while the same loading at 0.33 Hz could promote matrix catabolism without the involvement of any external cytokine [84,85]. The authors suggested that the response of human AF cells to cyclic tensile strain is frequency-dependent, affected by degeneration [84,85].

### 2.6. Genetic Influences on Disc Degeneration

IVD degeneration is a multifactorial, progressive disease, and recent evidence has also correlated disc degeneration with genetic mutations. Disc degeneration and herniation, for example, have been linked with a high genetic predisposition, which in some cases can have a hereditability of nearly 60% [86,87]. The most crucial genetic mutations are genes that code for ECM components, such as collagen and aggrecan [88,89,90]. Mutations of collagen type II (specifically COL2A1) led to highly disorganised ECM deposition in transgenic mice, along with the total absence of endochondral bone or the epiphyseal growth plate [88]. Similarly, a genetic mutation of collagen IX in a mouse model was associated with the shrinkage and disappearance of NP tissue and fissure formation in the AF, which are both early signs of disc degeneration [89]. Aggrecan, like collagen, is another important ECM component for IVD, and therefore, mutations of aggrecan-associated genes have been linked with degeneration. In a study by Watanabe et al., for example, it was shown that mouse cartilage deficiency known as ‘cmd’ was associated with a genetic defect of aggrecan synthesis, causing heterozygotes mice to have cervical spine misalignment and herniation, which is a cause of premature death [90]. On the other hand, single-nucleotide polymorphisms associated with ADAMTS-5, one of the major aggrecanases in the IVD, were associated with the formation of cracks and tears in the AF [91]. More recently, mutations and depletions of other genes, such as Paired box-1 (PAX1) and Forkhead box F1 (FOXF1), were also associated with spine deformations in the foetal disc [92,93]. Deletion of the FOXF1 gene in mice, for example, was associated with spinal deformations and fusion of vertebrae [92], while deletions of PAX1 were associated with aberrant vertebrae formation during axial skeleton development due to the key role played by PAX1 in sclerotome formation [94].

## 3. Impact and Current Treatments of Disc Degeneration

### 3.1. Socio-Economic Burden

Low back pain (LBP) is one of the most common degenerative diseases in Western countries, whose prevalence is increasing dramatically in recent decades and with the highest number of years lived with disabilities (YLDs) [95,96]. Although it is recognised that LBP is a multifactorial condition, 40% of cases affected have been associated with degeneration of the IVD [66]. Recently, de Schepper and co-workers, in a cross-sectional, population-based study, confirmed a strong association between LBP and IVD degeneration by finding a high prevalence of disc narrowing and osteophyte formation (two morphological features of disc degeneration) in people affected by LBP [97]. It has been estimated that nearly 84% of the world population will experience LBP during their lifetime, with the highest impact in Europe and the lowest impact in Africa, followed by South America and India [96,98]. The prevalence of LBP seems to increase steeply after the third decade of life, with a similar growth rate between men and women and with a peak over the age of 65 [99]. Among people affected by IVD degeneration, it has been assessed that at least 10% remain chronically disabled [99]. It has been estimated that in the UK alone, the socioeconomic burden of LBP costs around GBP 12 billion per year, while in the USA, the total cost associated with LBP has been estimated to be nearly USD 85 billion per year [95,100]. However, these estimates are conservative since LBP does not represent an economic burden only for the patient, but it has an enormous impact on carers, families, communities, and the healthcare system [101]. A deeper analysis of the socio-economic costs of LBP showed that indirect costs are often higher than direct medical costs. In Australia, for example, total costs for LBP are estimated at AUD 9 billion, where only 11% of the total amount accounts for direct costs. Similar amounts and proportions have also been reported for Netherlands and UK [96]. Moreover, due to the relation between ageing, spine overloading due to obesity, and LBP, it has been estimated that the total costs associated with LBP will increase steeply in future decades unless regenerative therapies to restore tissue function are implemented [99].

### 3.2. Grading Scales for the Assessment of Disc Degeneration

**Figure 4:**
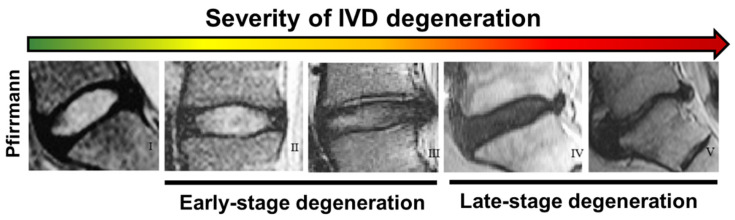
Comparison between different stages of intervertebral disc degeneration (**I** to **V**, left to right) according to the Pfirrmann grade scale based on MRI images. Reprinted and adapted with permission from Ref. [103]. Copyright 2022, Elsevier.

To date, magnetic resonance imaging (MRI) is the most used technique to clinically assess the onset of IVD degeneration associated with LBP. MRI is a powerful, non-invasive technique through which it is possible to obtain not only information about the disc height but also about the hydration level of the NP. Based on MRI images, in 2001, Pfirrmann and colleagues developed a disc-degeneration grading system able to estimate the water content signal in degenerated discs and correlate it with morphological parameters on a scale from I to V [102,103]. On MRI images, healthy discs appear bright for the NP and for the inner part of the AF due to their high level of hydration, while the outer AF appears dark. With the progression of IVD degeneration, the intensity of the NP is drastically reduced, and the distinction between the inner and outer AF is lost [102]. Taken together, the colour of the NP, demarcation between NP and AF, signal intensity, and disc height are used as morphological features to describe the grade. The Pfirrmann grading scale shows the changes in IVD degeneration from one stage to the other, with degeneration becoming more destructive as one moves from grade I to V (Table 1). In particular, grades I–III are usually associated with early degeneration, while grades IV–V are reported as late-stage or advanced disc degeneration [102] (Figure 4). Although the Pfirrmann grading scale has been widely accepted, it should be used carefully since it shows some inherent limitations. First, the grading system proved to be relatively non-discriminatory when elderly subjects were examined since, due to ageing, the NP becomes dehydrated, and the distinction between aged and degenerated discs becomes hard [104]. Second, the system is based on subjective and often ambiguous visual assessments of MRI images, so degenerated discs may be categorised into different levels of degeneration depending on the assessor. As such, very similar contiguous grades, such as grade III and grade IV, are difficult to distinguish, so highly degenerated discs may be underestimated [104]. Recently, Griffith et al. have slightly modified the five-level Pfirrmann grading scale into an eight-level scale to also discriminate IVDD severity in elderly subjects, where the Pfirrmann grading scale lacked accuracy [104]. Other parameters, such as the ‘MRI Index’, which is the product of the NP pixel area (or volume) with the intensity of the signal, are also being explored as more objective tools [105]. However, Pfirrmann’s grading scale results are the most widely accepted [106,107].

### 3.3. Conservative Therapies for Early Stage Disc Degeneration

Current treatments for IVD repair are divided into conservative and surgical therapies depending on the Pfirrmann grade of degeneration. For early stage degeneration (Pfirrmann grade < III), a series of conservative approaches is recommended before employing any invasive therapy. Conservative approaches are entitled ‘palliative care’ and can include, depending on the case, imposed rest, physical exercises to strengthen back muscles, physiotherapy, painkillers, and vitamin supplements [108]. Physical exercise has been shown to correlate positively with cell proliferation in several animal studies, while strengthening of the paraspinal muscles has been shown to have a positive effect on pain reduction [76,109]. Along with physical exercise, severe cases of LBP require the administration of non-steroidal anti-inflammatory drugs (NSAIDs, such as ibuprofen and paracetamol), corticosteroids, opioids, and muscle relaxants. These medications can be supplied via oral administration or via intradiscal injections, whose aim is to decrease the local inflammation and provide temporary anaesthesia to relieve the pain [108]. Among supplements, omega-3 fatty acids, glucosamine, and chondroitin are usually administered due to their minimal side effects, but there is a lack of evidence and conflicting findings on their therapeutic effect [110,111]. Although these treatments can offer temporary pain relief, these medications do not target the process of the disease (NP matrix degradation, NP cell senescence and death, or altered cell function), but only target the symptoms of LBP. Moreover, to ensure a therapeutic effect and overcome poor targeting, multiple doses are often required, without any guarantee of their sustained effect. In particular, NSAIDs, which are mainly based on COX inhibitors, can increase susceptibility to mucosal injury in the gastrointestinal tract, forcing the patients to take other medications, such as proton pump inhibitors, to cope with these complications [112,113,114].

### 3.4. Surgical Approaches for Late-Stage Disc Degeneration

When patients have not achieved pain relief after 6 months of non-surgical procedures and they are still physically constrained in daily activities, surgery is the only remedy [108]. Surgical operations are only performed to tackle late-stage disc degeneration (Pfirrmann grade > III) when disc function and mobility are irremediably compromised. Although different surgical procedures are currently available, their common goal is to separate nerve vessels from the disc by reducing the size of the disc bulging on the neural arch and restoring normal disc function [108,115]. Reducing disc bulging is often achieved through decompression or discectomy (mechanical, thermal, or chemical), in which a portion of IVD is removed to decompress the spinal canal and destroy the blood and nociceptive fibres that could have formed in the periphery of the disc during degeneration [108]. When the whole disc is compromised (Pfirrmann grade > IV), total disc replacement (TDR) and spinal fusions are the only options available to treat this disability. In TDR, the entire IVD (NP plus AF and CEPs) is substituted by an artificial implant made of polymers (for NP and AF bodies) and metals (for the CEP to anchor on the vertebral bodies) [116]. Commercially available products for TDR include InMotion/Charité^®^, ProDisc^®^, and Maverick^TM^, while BioDisc^TM^, GelStix^TM^, and PerQdisc^TM^ have recently been explored for NP replacement [117,118,119,120]. Metal and plastic materials are chosen because they are bioinert and, because of their high mechanical properties, they are suitable to support the biomechanics of the column. In spinal fusion, two or more vertebrae are merged into one functional unit [115]. During this procedure, vertebrae enclosing the damaged disc are fused together by the addition of tissue graft containing osteoinductive materials (e.g., bone allografts, autografts, and cements) to allow vertebrae fusion. Finally, the vertebral joint is stabilised through external fixators, such as surgical plates and screws [116].

The introduction of an inert biomaterial or the removal of a damaged disc is often a very invasive procedure accompanied by medical complications and poor efficacy in the long term. Inert biomaterials, in fact, are not living materials, so the damaged tissue is actually *substituted* rather than *repaired*. The incorporation of a solid implant can cause a mechanical mismatch between the implant and the adjacent vertebrae, which can result in hypermobility and the onset of IVDD in the adjacent discs [121]. Any abnormal process that occurs in the mobile segment adjacent to a spinal fusion is clinically defined as Adjacent Segment Disease (ASD). The list of abnormal processes occurring at the surrounding vertebrae and IVDs is long and includes herniated NPs, stenosis, hypertrophy of facet joints, and instability of the column [122]. From the several retrospective clinical reports that have been published on ASD, it emerged that the incidence of this medical complication is broad, ranging from 7% up to 52% [122]. In most cases, as reported by Leong [123], Guigui [124] and more recently by Kuslich’s groups [125], ASD causes degenerative changes of the IVDs next to fused segments. In general, when post-fusion patients present pathological symptoms referable to ASD, conservative approaches are used to relieve the pain, but in most cases, another surgical intervention is required. Kim et al., for example, in a follow up of 1856 patients who underwent surgery for degenerated IVDs, reported a cumulative recurrence rate of up to 16% in 10 years [108]. Moreover, these implants require periodic revisions and are often accompanied by local infections arising near the fixators (surgical plates or bone screws), which may force the patient to undergo a second surgical operation [126].

### 3.5. Cell-Based Therapies for NP Regeneration: Cell Sources

As stated in the previous section, commercially available IVD or NP replacement materials are poorly efficient in the long term since they partially restore column segment mobility and do not biologically repair or regenerate the affected tissue. Moreover, they are often accompanied by a broad range of potential adverse effects and a high rate of recurrence. Hence, it is critical to develop novel therapeutic strategies that can promote IVD regeneration and can be translated into clinics to improve patients’ quality of life. Since IVD degeneration initiates in the nucleus, the NP represents a promising tissue target for the design of novel therapies. In particular, tissue engineering (TE) and regenerative medicine can offer potential alternative therapies to current treatments by the use of instructive and functional biomaterials in conjunction with cells and biomolecules (e.g., GFs) as therapeutic agents. Indeed, biomaterials can help to restore disc height and IVD biomechanics as well as provide a vehicle for the retention and delivery of cells and relevant biomolecules to injured discs to re-establish healthy tissue.

The use of cells as therapeutic agents, with or without encapsulation in a biomaterial, is referred to as cell-based therapy. Cell-based approaches may involve the delivery of autologous, allogeneic, or xenogeneic sources of primary or stem cells, which can be injected intravenously, transplanted at a defect site within a scaffold, or recruited from a patient’s own tissue to exploit the self-repair processes [127]. Different cell sources and cell types have been explored for cell-based therapies. Autologous cells are usually preferred since they are harvested from the patient’s body to avoid transplant rejection. Mesenchymal stem cells (MSCs), for example, are receiving a significant amount of attention for the treatment of NP defects since they have been shown to undergo differentiation into NP-like cells, with high expression and deposition of proteins specific to the NP matrix [128]. Moreover, MSCs can be isolated from a large number of adult connective tissues, including bone marrow [129], umbilical cord [130], trabecular bone [131], adipose tissue [132], dermis [133], skeletal muscle [134], and AC [135]. In particular, the possibility to acquire MSCs from tissues such as fat or skin has limited the morbidities associated with stem cells harvesting, making cell isolation less invasive.

Recently, Clarke and co-workers tested the suitability of bone-marrow-derived MSCs (BM-MSCs) and adipose-derived MSCs (AD-MSCs) in conjunction with GDF-6 and TGF-β3 for the differentiation into NP-like cells for the treatment of IVD degeneration [136]. The authors found that GDF-6 stimulation of both types of stem cells induced a high expression of NP-marker genes and GAG production, with a higher effect using AD-MSCs rather than BM-MSCs. Moreover, GDF-6-stimulated AD-MSCs resulted in a softer ECM deposition, more akin to the native NP tissue [136]. MSCs are also explored for their ability to release bioactive factors and anti-inflammatory agents that can influence tissue regeneration and reduce the immune response and local inflammation. Moreover, cell-to-cell contact between MSCs and endogenous primary cells has shown to be beneficial for cell differentiation and ECM production in co-culture studies, suggesting that the injection of MSCs in degenerated discs may also be beneficial for resident NP cells [128]. For example, in a study by Strassburg et al. the co-culture of MSCs and NP-cells increased the expression of anabolic GFs by MSCs, differentiation towards the discogenic lineage, and improvement of the NP cell phenotype through MSCs-NP cell communication, which was possibly mediated by the bi-directional intercellular transfer of membrane components [137,138]. Taken together, these features confer MSCs’ high clinical translatability for degenerated discs.

As shown recently by a systematic review by Panebianco et al., along with MSCs (59.5% of peer-reviewed articles screened), NP cells (40.5% of articles screened), patient or animal-derived, have also been employed in clinical studies and in preliminary in vitro studies for NP repair [139]. The transplantation of NP cells, for example, has been shown to retard the degenerative disease in a dog model, whereas a significant decrease in LBP score with the retention of disc height and hydration levels was observed in a randomised human clinical trial [140,141]. Despite these positive results, the senescent and catabolic phenotype of autologous cells harvested from degenerated discs introduces doubts to its use for cell therapies, in which normal or appropriate cell function is a strict requirement. Moreover, the acquisition of these cells may lead to further complications that can accelerate degeneration [142]. Nevertheless, the use of NP cells extracted from animal models is strongly encouraged when they are tested in conjunction with novel biomaterials, whose properties of biocompatibility and cell response are still unknown. In particular, many animal-sourced NP cells are derived from preclinical models of IVD degeneration widely accepted in the literature, which enables rapid progression from in vitro to in vivo testing [143]. Among the animal sources used for NP repair strategies, rabbit NP cells are the most commonly used (34.4%), followed by larger animals such as cows (25.5%) and pigs (21.1%) [139]. Bovine and porcine NP cells are largely preferred because they are cost-effective, and they are ideal for simulating in vitro force applications and transport studies, similar to those experienced by the human IVD [144].

### 3.6. Cell-Based Therapies for NP Regeneration: Biomaterial Requirements

For the biomaterial design aspect, biomaterials should fulfil specific requirements, which can be divided into ‘mechanical’, ‘biological’, and ‘clinical’. Although the biomaterial should match the mechanical properties of the native NP on the one hand, on the other hand, it should be applicable and translatable into a clinical setting to be effectively used as an alternative therapy to current treatments.

*Mechanically* speaking, the biomaterial should restore the disc height and ensure biomechanical flexibility. To meet this requirement, the biomaterial should possess rheological properties similar to those of the NP, such as shear modulus, swelling behaviour, and permeability. As detailed by Iatridis et al., the NP tissue is a gelatinous material, with an average storage modulus at 1 Hz (*G*′, accounting for the material’s elasticity) of 10 kPa and a loss modulus (*G*″, accounting for the material’s viscous properties) of 4 kPa [145]. Swelling properties are also important since the NP will swell over time under cyclic loading, and this is usually reported as a ~1.5% change of its weight for the human NP [146]. NP-mimicking biomaterials should also have values of permeability (*k_a_*) similar to those of the human NP (*k_a_* = 6 × 10^−16^ m^4^ N s) to ensure a physiological exchange of nutrients and waste with the surrounding tissues, i.e., the AF and the CEP [146]. In addition, the biomaterial should be hydrophilic and highly hydrated to mimic the high water content of the native NP tissue (water content: 80–90%) [7].

*Biologically* speaking, ideally, the biomaterial should be able to inhibit the degenerative processes, prevent the formation of nerve and blood vessels, and stimulate tissue regeneration. To this extent, cell-based therapies in which the biomaterial is used as a vehicle for cells, proteins, and/or GFs has proved to be a successful strategy in numerous preclinical and clinical studies for NP repair [147,148]. Indeed, the incorporation of exogenous cells into an implanted biomaterial can promote ECM synthesis as well as secrete paracrine signals that may stimulate resident cells to deposit new ECM and release immunomodulatory signals to halt disc inflammation [149]. In line with Langer and Vacanti’s principles of TE [150], to be biologically successful, biomaterials designed as cell carriers should retain implanted cells at the injury site and provide them with an instructive microenvironment that can support cell viability, proliferation, and differentiation [151]. At the same time, biomaterials should allow tissue remodelling, with a degradation rate that ideally should match the rate of tissue ingrowth during regeneration [151].

*Clinically* speaking, the designed biomaterial should be deliverable via minimally invasive tools (e.g., small-needle syringes, catheters) and be non-immunogenic in vivo in order to interdigitate and fully integrate with the NP ECM after implantation [152].

## 4. Injectable Hydrogels for NP Regeneration

### 4.1. Advantages of Using Injectable Hydrogels for NP TE

Hydrogels and, in particular, injectable ones, represent an ideal class of biomaterials to serve as cell carriers and/or as acellular scaffolds for NP TE for several reasons. Firstly, hydrogels are hydrated materials with a gelatinous and amorphous structure that closely mimics the viscosity and level of hydration of the NP. Secondly, hydrogels can be designed to be injected via minimally invasive techniques, such as syringe injections and catheters. With this approach, hydrogels can easily reach the NP, which is known to be avascular and therefore difficult to reach systemically. Hydrogels can be injected via small punctures (e.g., 26G–21G needles), which reduces the damage occurring via annular puncture to a minimum and allows faster recovery after the injection [151]. Due to their viscoelastic properties, hydrogels can flow easily and interdigitate within the NP to fill tissue defects and fissures. Moreover, injectable hydrogels can be designed to incorporate a range of biophysical and biochemical cues (e.g., GFs or anti-inflammatory cytokines) so that they can be used at different stages of disc degeneration. Indeed, hydrogels may be applicable to symptomatic early to moderate disc degeneration (Pfirrmann grade < 3) to attempt regeneration, while they may also be used as a prophylactic approach in segments adjacent to the degenerated discs of patients undergoing disc fusion to prevent the onset of adjacent vertebrae’s degeneration [122].

Finally, hydrogels can serve as protection to retain their cargo (e.g., cells and/or biomolecules) at the injury site to avoid its leakage as well as to protect it against harsh environments. Degenerated discs are an example of such harsh microenvironments, being full of catabolic cytokines, with minimum levels of glucose, low oxygen, and acidic pH (pH < 6.2) [46,48]. Direct injection of cells without any cell carrier is usually accompanied by poor engraftment at the defect site (less than 3% of the total transplanted cells) along with a loss of control over cell fate after transplantation [153]. In a study by Vadalà et al., for example, injections of MSCs into IVD without any cell carrier were accompanied by cell leakage from the injection site, with leaked stem cells being involved in the formation of osteophytes on the vertebral bodies [154]. Similarly, it has been observed in vitro that MSC proliferation and matrix deposition is severely compromised when cells are exposed directly to low glucose, high osmolality, and acidic pH, which represent the environmental conditions of degenerated discs [155].

Due to their properties, injectable hydrogels exploited as cell carriers can represent an alternative therapy to current treatments by bridging the gap between mechanical (provided so far by disc prostheses) and biological needs (provided by cell transplants). Once injected, hydrogels may be used to provide high initial biomechanical efficiency to degenerated discs in the short term while still promoting tissue repair in the long term.

### 4.2. Naturally Derived Injectable Hydrogels

Based on the origin of their building blocks, hydrogels can be classified into natural (or naturally sourced) and synthetic. Most natural hydrogels used for IVD repair include hyaluronic acid, alginate, and chitosan [156]. These hydrogels are thought to have an advantage over synthetic counterparts since their building blocks are naturally presented *in vivo*, showing intrinsic ECM mimicry and inherent biocompatibility. Hyaluronic acid (HA) is an unbranched high-molecular-weight polysaccharide existing in the native ECM of most hydrated tissues, such as AC, synovium, and IVD, where it plays a key role in maintaining high levels of water content [157]. When dissolved in water, HA can create highly viscous solutions, which are also used in clinics as injectable viscosupplement to replenish the human synovium of HA during osteoarthritis [158]. For IVD research, Pandit’s group showed that cross-linked HA hydrogels were able to induce a downregulation of inflammatory receptors and neurotrophins in NP cells exposed to IL-1β in an in vitro inflammation model [159]. The exact mechanism of HA seems to be related to the binding of HA chains to the cells’ CD44 receptor, which prevents NP cells from undergoing further inflammation [159]. Similarly, injection of MSC-laden 15% HA hydrogels in rat IVDs showed cell proliferation and an increase in disc height, as well as a reduction in pain and inflammation [160]. However, HA is prone to undergoing rapid hydrolytic degradation *in vivo*, which results in generally poor mechanical properties [161]. Moreover, degradation of HA in small fragments has been shown to induce mRNA expression of key catabolic and pro-inflammatory cytokines by IVD cells, which is not clinically preferred for the treatment of degenerated discs [162].

Along with HA, alginate represents another popular material choice for NP TE. Alginate is a polysaccharide extracted from marine algae, with high biocompatibility and low cytotoxicity. Unfortunately, the applications of alginate hydrogels in the field of IVD repair are limited so far due to alginate’s physiochemical properties, such as long gelation times and high degradability. Alginate monomers undergo gelation when exposed to divalent calcium ions that induce ‘eggbox’ chelation of its monomers, mannuronic and glucuronic acid. However, alginate’s gelation is a quick process, during which the hydrogel boundaries bond tightly while the rest of the hydrogel remains physically weak. In a study by Kalaf et al., for example, ‘slowly cross-linked’ alginate gels were implanted in an ex vivo bovine IVD model and showed the ability to maintain disc height over cyclic axial loads better than alginate hydrogels that were ‘instantaneously gelled’ [163]. The elution of calcium ions over time with a decrease in mechanical properties is another major drawback of this system. Baer and colleagues, for example, showed that although AF and NP cells cultured in alginate gels were able to express collagen type I and II, respectively, the mechanical properties of the cell-laden hydrogel constructs decreased over 21 days, up to 50% of their initial strength [164].

Like alginate, chitosan has also been used extensively in preliminary studies of NP TE, but its long gelation time and weak mechanical properties make it poorly applicable as an injectable hydrogel for IVD repair. Chitosan hydrogels are formed by cationic polymeric chains of chitin, which is largely abundant in the shells of crustaceans and in the wings of arthropods. Chitosan has excellent biodegradability, water dispersibility, promotes cell adhesion, and its cationic chains can be exploited to retain negatively charged PGs in the hydrogel network [156]. Chitosan can be mixed with beta-glycerophosphate (BCP) to make its gelation thermosensitive and faster so that chitosan hydrogels can be injected into degenerated discs and undergo gelation in situ [165]. Unfortunately, although high levels of BCP are beneficial for chitosan’s gelation, BCP in high concentrations has been revealed to be highly cytotoxic [166]. Alini’s group, for example, successfully cultured bovine NP cells in chitosan gel and showed a high retention of NP-synthesised PGs within the hydrogel, but the gel became cytotoxic when exposed to AF cells [167]. In addition, chitosan gels present relatively low mechanical properties and poor cell adhesion, which are not ideal for load-bearing tissues, such as the NP [156].

Other natural hydrogels, such as fibrin, gelatin, cellulose, and gellan gum, have also been explored as IVD injectable systems. However, as occurs for HA, alginate, and chitosan, typical natural hydrogels show poor mechanical properties, a high degradation rate, and low adhesion properties, so extensive cross-linking or co-assembly with another hydrogel network is usually required to overcome these issues [156]. In some cases, hydrogel cross-linking approaches involve the use of chemicals (e.g., glutaraldehyde and glyceraldehyde) that are highly cytotoxic and whose by-products should be removed completely before cell encapsulation, while some cross-linking mechanisms rely on UV-photocurable materials, which is not practical for NP injection [168]. Finally, due to their animal or vegetable source, many natural hydrogels suffer from inherent batch-to-batch variability, which makes them poorly reproducible for clinical studies [169].

### 4.3. Synthetic Injectable Hydrogels

Some of the limits encountered with natural hydrogels, such as batch-to-batch variability, low mechanical properties, and high degradation rates, can be overcome with the use of purely synthetic materials. Indeed, synthetic hydrogels allow easier large-scale production, high consistency across batches, and highly tuneable properties. Among synthetic injectable hydrogels used as cell-laden scaffolds for NP repair, poly(N-isopropylacrylamide) (pNIPAM), poly(ethylene glycol) (PEG), and poly(vinyl alcohol) (PVA) hydrogels are the most used in the field [156]. PNIPAM hydrogels have a thermosensitive network with hydrophilic and hydrophobic building blocks. When the temperature of the pNIPAM solution is below its lower critical solution temperature (LCST), the hydrophilic building blocks of this polymer interact with water, and they are able to form a cross-linked hydrated network. Once the temperature is raised above the LCST, the hydrophilic building blocks dehydrate and collapse into a globular system. The transition temperature between the two states can be set to be the human body temperature, allowing pNIPAM to undergo gelation in situ once injected. To this extent, Le Maitre and Sammon’s groups developed a pNIPAM-based hydrogel that is liquid ex vivo at 39 °C while undergoing gelation in vivo at 37 °C, triggered by body temperature, when it is injected into the IVD [170]. The authors showed that a pNIPAM-clay hydrogel composite promoted the differentiation of MSCs into NP-like cells without additional GFs in hypoxic conditions mimicking degenerated discs [171]. However, the biggest disadvantage of this system is that pNIPAM hydrogels are not degradable; therefore, after gelation, cells are entrapped in the hydrogel and tissue remodelling by encapsulated cells may be hindered [172].

Due to its biocompatibility, non-immunogenicity, and ease of functionalisation, PEG hydrogels are widely studied in TE and for NP repair. However, due to the lack of their recognition site by encapsulated cells and non-degradability, PEG is largely used either as an interpenetrating network in hydrogel composites or as a non-toxic hydrogel cross-linker. Similarly, PVA hydrogels are usually based on composite mixtures of PVA and other polymeric matrices, either natural (e.g., silk fibroin, cellulose) or synthetic (e.g., poly(vinyl pyrrolidone) or PVP) [156]. PVA-based hydrogels, like other polyesters, start to degrade when hydrolytically labile linkages are exposed to water; therefore, the degradation rate and time of the final hydrogel can be tuned for specific applications and environments. However, the use of polyesters is not recommended for the repair of degenerated discs since hydrogel degradation leads to the formation of acidic by-products that cause further acidification of the hydrogel at the injection site. Moreover, acidic by-products have also been shown to have an ‘auto-catalytic’ effect on hydrogel degradation [173]. In the case of degenerated NP, where the pH can go down to 6.5, further acidification at the injection site would be deleterious for the resident cells, promoting a catabolic phenotype.

Despite the several advantages of synthetic hydrogels, their lack of biocompatibility and the need for chemical cross-linking with toxic reagents represent issues that need to be overcome before any biological translation. In particular, although many synthetic hydrogels show excellent mechanical properties and ease of injectability, these systems lack biological cues that could promote cell adhesion and proliferation. In many cases, in fact, synthetic hydrogels are coated with ECM proteins or decorated with corresponding biological motifs for TE applications [174,175]. Table 2 summarises the major advantages and disadvantages of natural and synthetic hydrogels.

## 5. Hydrogels Based on Self-Assembling Peptides

A versatile class of hydrogels that combine the advantages of both natural and synthetic hydrogels is represented by self-assembling peptide hydrogels (SAPHs). Peptide hydrogels offer great potential for their use as a 3D scaffold and 3D cell carriers for NP TE since they satisfy most of the criteria cited above. These systems undergo a sol–gel transition in hydrophilic environments through the mechanism of molecular self-assembly, without the need for any toxic cross-linkers that are usually needed for natural and synthetic hydrogels [176]. Molecular self-assembly, in fact, is a kinetically and thermodynamically driven, bottom-up approach, which is ubiquitous in nature, allowing the formation of different structures from nanoparticles to nanofibres and supramolecular constructs present in biology, such as virus capsids and ribosomal units [177].

SAPHs are water-rich (>95%) and have a nanofibrous microenvironment that mimics the native ECM and creates a suitable platform for cell encapsulation and 3D culture [176,178]. Moreover, they can be designed to show specific peptide length and bioactive motifs and affect the network elasticity, which can be exploited as a physiochemical regulator of cellular fate [179]. Along with their biocompatibility, peptide hydrogels are also shear-thinning and are easily injected via syringes or catheters, with the ability to recover their bulk properties after injection/transplant, which makes them an ideal candidate for minimally invasive therapies [180]. The majority of SAPHs are based on L-amino acids, which are naturally found and processed by the human body, making them low in terms of immunogenicity and inflammatory properties [181,182]. Peptides are typically synthesised using fluorenylmethoxycarbonyl (Fmoc) solid-phase synthesis, which results in the production of short peptide sequences with high purity (>95%) [183]. This high purity makes peptides more reproducible and reduces the batch-to-batch variability encountered with naturally derived hydrogels. Indeed, many peptide systems are now readily available in the market, including PuraMatrix (from Corning, US), HydroMatrix (from Sigma-Aldrich, UK), and PeptiGels (from Manchester BIOGEL, UK), which highlights the ability of these systems to be scaled up for large production. Moreover, some SAPHs have also started to find their way into approved clinical products over the last decade [184].

### 5.1. Amino Acids as Molecular Building Blocks

**Figure 5:**
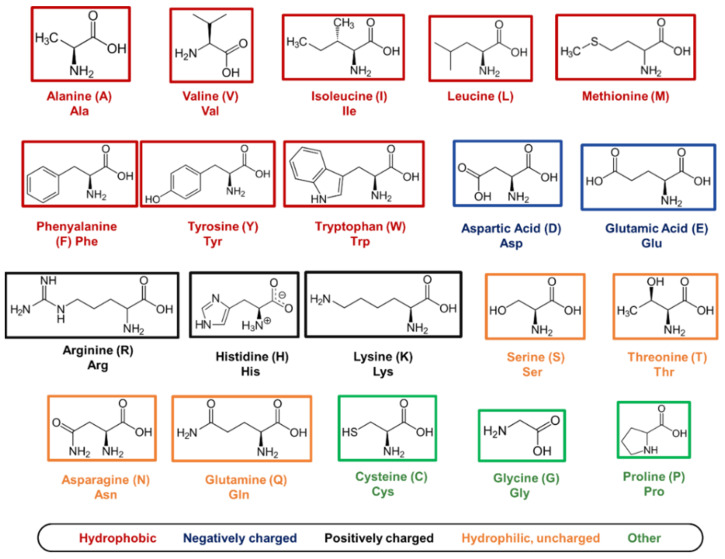
List of 20 natural amino acids. For each amino acid, its 3-letter abbreviation, 1-letter code, and property (‘hydrophobic’, ‘negatively charged’, ‘positively charged’, ‘hydrophilic, uncharged’, and ‘other’) are provided. Cys, Gly, and Pro are listed as ‘other’ since they have specific roles in peptide self-assembly that cannot be associated with the other properties listed.

At the base of molecular self-assembly, there is the precise choice of building blocks, which will adopt the most energetically favourable conformation to produce highly organised structures with defined properties. In nature, twenty amino acids are available as building blocks for the synthesis of peptides and proteins. All amino acids, except for glycine (G), are chiral macromolecules that exist in biology in their L-form, with a common structure consisting of a central carbon atom (alpha carbon) covalently bound to a carbonyl (-COOH) and an amine group (-NH2), along with a specific R-group. The configuration of a peptide sequence strongly depends on the nature of the R-groups that are present between two adjacent amino acids. Indeed, according to the properties of the R-group, amino acids can be divided into hydrophobic, hydrophilic, charged, and ‘others’ [185] (Figure 5). Amino acids interact with each other in the same peptide sequence or with adjacent peptides through a vast range of physiochemical interactions, including H-bonding, ionic bonds, pi–pi stacking, and electrostatic and hydrophobic interactions. Typically, aromatic and hydrophobic amino acids are involved in pi–pi stacking interactions, while hydrophilic residues are involved in charge–charge interactions and H-bonding [185]. Inversely, cysteine is typically exploited for chemical functionalisation, glycine to add peptide flexibility, while proline is used for chemical hindrance [185].

As will be discussed in the following sections, during peptide synthesis, amino acids are linked together one after the other in a linear chain, and the position of amino acids plays a key role in both peptide self-assembly and in the interaction of peptides with the surrounding environment [178]. Using a bottom-up approach, amino acids can be exploited to recreate secondary and tertiary structures observed in cellular proteins. A linear chain of amino acids (or peptides) represents the primary structure of amino acids, while secondary and tertiary structures describe how peptide chains are folded into a 3D conformation. Typically, the length of a peptides chain is usually reported as between 2 (dipeptides) and <50 amino acids (polypeptides). Depending on the constituent amino acids, peptides acquire secondary structures in solution, such as β-sheets, β-hairpins, α-helices, and random coils, which can further self-assemble into supramolecular structures in response to a change in ionic strength, pH shift, enzymes’ activation, peptide concentration, light, and temperature [185]. SAPHs are, therefore, the result of secondary structures assembling/entangling over a critical gelation concentration into self-supporting water-swollen networks, i.e., 3D hydrogels [186]. According to the secondary structures acquired, SAPHs are divided into nature-mimicking structures (e.g., α-helices and β-sheets) and newly designed derivatives, such as amphiphilic and short aromatic peptides.

### 5.2. Self-Assembling Peptide Structures: β-Sheets and β-Hairpins

**Figure 6:**
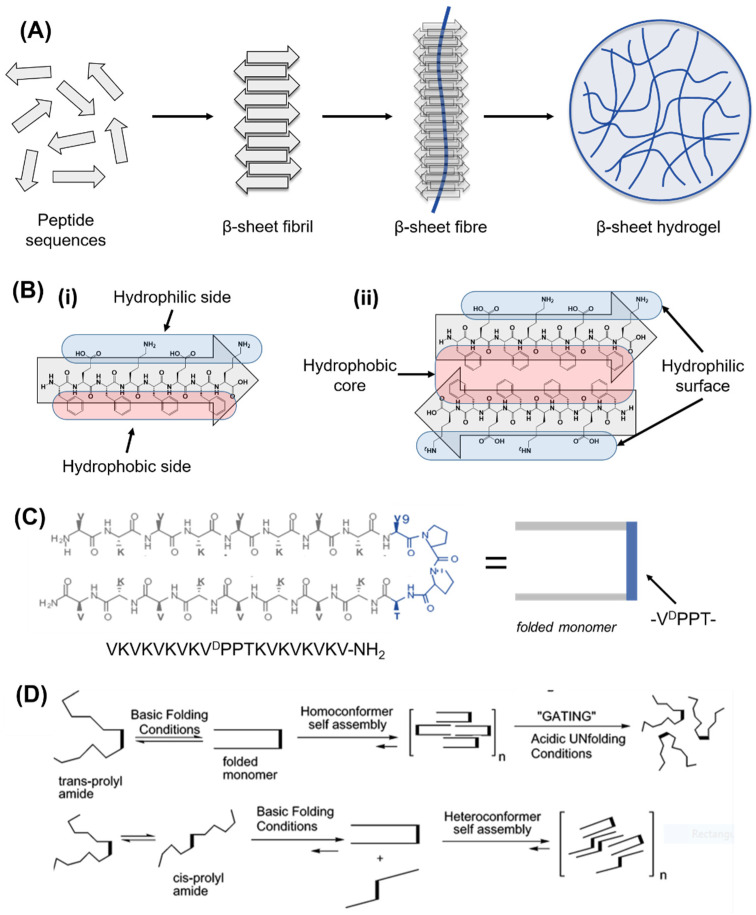
Beta-sheet and beta-hairpin peptide system designs. (**A**) Schematic representation of β-sheet-forming peptide hydrogel formation. Peptide sequences under external stimuli (e.g., pH, enzyme, temperature, light, time) self-assemble into β-sheet fibrils and fibres, which above a critical gelation concentration entrap water in water-swollen networks, i.e., hydrogels. (**B**) Detail of a β-sheet-forming polypeptide (**i**) reacting in water with another polypeptide via stacking of hydrophobic regions (**ii**). (**C**) Design of a β-hairpin sequence (i.e., MAX1), in which two valine-based peptides are linked together by a -V^D^PPT- turn. This tetrapeptide based on D-isomer valine induces a trans-prolyl amide bond re-arrangement favouring the β-hairpin formation. (**D**) Folding/self-assembly pathways of β-hairpins. Adapted with permission from Ref. [200]. Copyright 2022, American Chemical Society.

The first example of SAPHs was introduced in 1989 by Zhang et al., who discovered that a peptide sequence of zuotin (AEAEAKAKAEAEAKAK or EAK16), a left-handed Z-DNA binding protein found in yeast, was able to self-assemble into nanofibres and form hydrogel networks in the presence of electrolytes. Inspired by this discovery, the first two sequences of complementary ionic polypeptides (16 amino acids), EAK16, and RADARADARADARADA (RADA16), were designed [184]. These β-sheet-rich fibrillar matrices proved to be highly biocompatible for 2D and 3D cell cultures of different cell lines [184]. Zhang’s discovery opened the route for the design of β-sheet forming peptides, in which charged hydrophilic amino acids are alternated with hydrophobic residues to form β-sheets units, which display one hydrophilic side and one hydrophobic site. When β-sheets are exposed to a hydrophilic environment, upon external stimuli, such as pH, temperature, enzymes, or light, the hydrophobic sides stack together, leading to the formation of different supramolecular structures, such as tapes, ribbons, fibrils, and fibres (Figure 6A,B) depending on the peptide concentration [185,186]. RADA16 and EAK16 have been extensively used in TE, both as pure peptides as well as combined with bioactive motifs, enzymes, and GFs. Bioactive motifs introduced in RADA16 systems include, but are not limited to, integrin-binding motifs (e.g., RGDS) and laminin-mimetic motifs (e.g., IKVAV) for enhanced cell adhesion and migration, VEGF-mimetic for angiogenesis, and BDNF-mimetic for neurogenesis [187]. Along with RADA16 and EAK16, dodecapeptides, such as KLD12 (KLDLKLDLKLDL), have also proved to self-assemble into fibrillar hydrogels with high biocompatibility and anti-microbial properties, which have been used extensively for cartilage, bone, and IVD applications [188,189,190,191].

In the last two decades, based on Zhang’s design, Saiani’s group developed a class of de novo β-sheet forming peptides based on short sequences (8 to 10 amino acids), which were able to entangle/assemble into nanofibrillar 3D network (fibres being 3–5 nm) and self-supporting hydrogels [192]. One of the earliest studies on these materials explored four octapeptides based on the alternation of alanine (A) and phenylalanine (F) with glutamic acid (E) and lysine (K), namely, AEAEAKAK, AEAKAEAK, FEFEFKFK, and FEFKFEFK. In that study, Saiani et al. showed that F-based octapeptides were able to form defined β-sheet fibres (~3 nm thick) and 3D fibrillar hydrogels regardless of the position of E and K in the sequence, while within the A-based systems, only AEAEAKAK self-assembled into rigid aggregated fibres [192]. From that study, different phenylalanine-based sequences of 8 to 10 amino acids were formulated and proved to be highly biocompatible, injectable, and low in terms of immunogenicity for a wide range of biomedical applications. Indeed, many cell lines were explored within this system for different biomedical applications, both in 2D and 3D settings, including osteoblasts [193], chondrocytes [194], NP cells [195], cardiomyocytes [196], and synoviocytes [197]. Moreover, this system is highly shear-thinning and able to recover after different cycles of shear strain, providing a versatile material platform for printable, injectable, and sprayable strategies [197,198,199]. During the self-assembly of β-sheets, two or more monomers, called β-strands, are joined together by at least two or three backbone H-bonds to form a twisted, pleated sheet. β-strands can join as parallel strands oriented in the same direction, such as N- to C-terminus, to form parallel β-sheets, or they can join in opposite directions, forming anti-parallel β-sheets. Based on this structure, Schneider and Pochan created a new class of peptide hydrogels by linking together two anti-parallel β-sheets via a β-turn to form a β-hairpin. In 2002, the first β-hairpin-based hydrogels for biomedical applications were formulated, namely, MAX1 and MAX8, in which a proline-based tetra peptide (-V^D^PPT-) was designed as a β-turn link to induce a type II’ turn structure [200] (Figure 6C,D). These hydrogels have gained interest due to their biocompatibility, their shear-thinning behaviour, and good mechanical properties [201]. Within these systems, in fact, several cell types (e.g., osteosarcoma cells, human trabecular cells, and fibroblasts) and biomolecules/drugs (e.g., curcumin, NGF, BDNF, and vincristine) were encapsulated and delivered successfully [202,203].

### 5.3. Self-Assembling Peptide Structures: α-Helices and Coiled Coils

**Figure 7:**
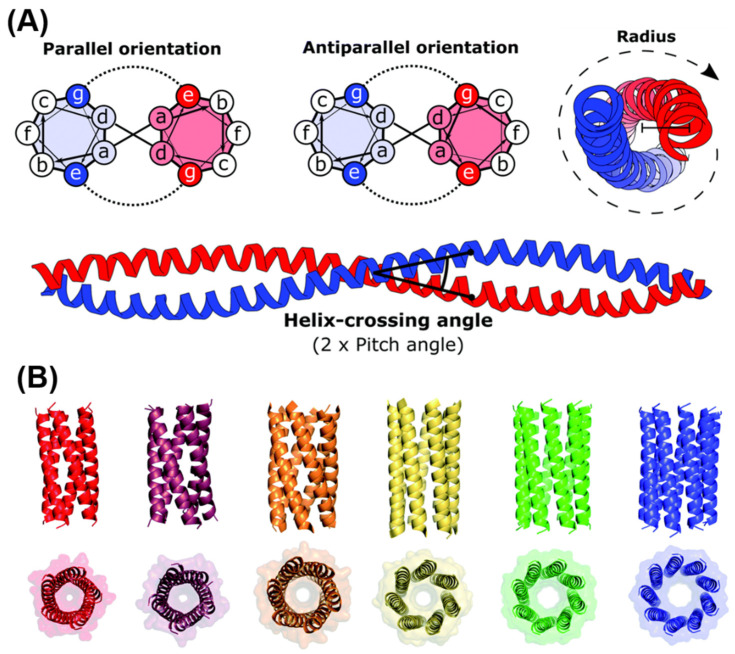
Coiled-coil and α-helix barrel peptide system designs. (**A**) Heptad wheel representation of parallel and anti-parallel coiled coils. Supramolecular structures form due to the interactions occurring between ‘a’ and ‘d’ (usually hydrophobic), while ‘g’ and ‘e’ (usually charged) stabilise the assembly. (**B**) Larger coiled-coil structures form α-helical barrels (αHBs) with accessible central channels. From right (red) to left (blue), X-ray crystal structures of multiple pentameric to nonameric αHBs. Images were adapted with permission from Ref. [206]. Copyright 2022, Royal Society of Chemistry.

An alternative to the use of β-sheets as a secondary structure to form fibrous hydrogels is provided by the α-helices, which are used as building blocks to form coiled-coil structures, as was proposed initially by Pauling and Corey [204]. The design of these structures relies on seven coil residues (*abcdefg* heptad), which possess a hydrophobic and a hydrophilic face according to the residues presented within the helix structure. In particular, the carbonyl group of the first amino acid of the heptad interacts with the fourth amino acid of the same heptad so that there are two turns within the same heptad [185]. A coiled-coil structure is the result of the interactions of two α-helices burying their hydrophobic sides within a hydrophilic environment (Figure 7A). The design rules of coiled-coil structures have been extensively studied by Woolfson’s group and are well understood [205]. Typically, *a* and *d* residues are hydrophobic, providing the hydrophobic core of the coil; *e* and *g* are generally charged amino acids (with opposite charge) used to stabilise the coil formation, while the triad *bfc* can be used to introduce bioactive motifs [185,206]. Examples of helical coiled-coil fibres are also present in numerous natural proteins, such as myosin and tropomyosin, fibrin, and keratin [207]. Variations of these systems are based on coiled coils with ‘sticky ends’, which stack together in a staggered manner along the axis of the coils. Larger coiled-coil systems can also form α-helical barrels with accessible channels, appealing as functional materials for the catalysis, binding, and transport of molecules (Figure 7B) [208]. These systems, as demonstrated by Woolfson’s group, offer a valid platform for regenerative medicine applications [209]. For example, Mehrban et al. showed that RGDS-decorated coiled-coil peptide hydrogels were able to sustain cell growth and differentiation of murine embryonic neural stem cells in an in vitro study [210]. The same system injected into a rat with a partial-thickness abdominal wall defect showed no foreign body reaction and good biocompatibility [211].

### 5.4. Short Aromatic Peptides and Peptide Amphiphiles

**Figure 8:**
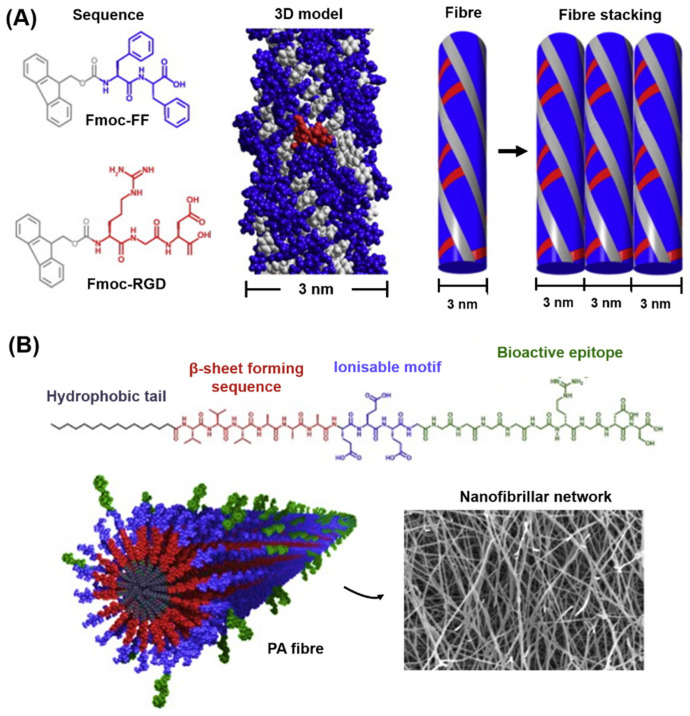
Examples of short aromatic and peptide amphiphile designs. (**A**) Chemical structures of Fmoc-FF and Fmoc-RGD assembling into nanofibrils with RGD sequences on the fibre surface. (**B**) Example of a PA sequence consisting of a hydrophobic tail, a β-forming segment, an ionisable motif, and a hydrophilic bioactive epitope. Self-assembled PAs show bioactive epitope on the surface (green appendages) and hydrophobic tails in the core (dark grey). Reprinted and adapted with permission from Ref. [223]. Copyright 2022, Elsevier.

Short aromatic peptide derivatives have been designed to create hydrogels based on π–π stacking interactions. In these systems, short peptides (typically up to five amino acids) exploit the presence of aromatic residues such as phenylalanine (F), tyrosine (Y), and/or tryptophan (W) as sticky points to promote self-assembly. The first example of short aromatic peptides was reported by Reches and Gazit, who found that diluting a hexafluoroisopropanol solution of Fmoc-FF in water caused self-assembly into amyloid-like fibrillar structures [212]. Since then, most common designs have been based on di-phenylalanine residues (FF) or di-phenylalanine coupled with Fmoc groups, which can also be extended to contain bioactive motifs such as RGD and serine residues (S) [185]. Fmoc-FF-based systems allow the creation of stable β-sheet-forming hydrogels in which Fmoc acts as an interlocking zipper to bring together different sheets along the nanofibre axis. In particular, the supramolecular unit of Fmoc-FF is thought to be composed of anti-parallel β-sheets organised into cylindrical fibres, held together by π–π stacking of adjacent sheets [185,213]. Two remarkable examples in this field are those by Jayawarna and Zhou, who designed FF-modified peptides able to create ECM-mimicking nanofibrous structures for the encapsulation of chondrocytes and fibroblasts in 3D cell cultures [214,215]. In particular, Zhou et al. showed that a mixture of Fmoc-RGD and Fmoc-FF self-assembled into RGD-displaying peptides that were able to promote fibroblast viability and provide a highly hydrated, stiff nanofibrous network [215] (Figure 8A). Jayawarna et al. extended the library of Fmoc-FF hydrogels by introducing serine (S), lysine (K), and aspartic acid (D) to form bioactive hydrogels for different cell lines [216].

Peptide amphiphiles (PAs) are represented by polypeptides with both hydrophobic and hydrophilic ends. Each PA sequence is composed of three parts: (1) a hydrophobic alkyl tail, (2) a β-sheet forming segment, and (3) a hydrophilic peptide head [185] (see Figure 8B). When exposed to water, PA sequences assemble into high-aspect-ratio rods having a hydrophobic core composed of the alkyl tails and a hydrophilic surface provided by the PA’s head. The glycine linker region is used to promote H bonding between adjacent PA monomers assembling within the same rod. By varying the alkyl tail length (12 to 16 carbon atoms), it is possible to change the fibre diameter, their flexibility, and, in turn, the PA’s network mechanical properties [185]. The peptide sequences of the hydrophilic head can be designed to show bioactive motifs to mimic the native ECM. This design strategy allows functional groups, such as cell attachment or GF-binding sites, to be ‘micro-patterned’ directly on the surface of peptide nanofibres, which could be used to regulate cell behaviour [217,218]. The possibility to introduce ECM-mimetic sequences directly exposed on the surface of the PA fibres without disrupting the process of self-assembly (which happens through the hydrophobic tail) represents one of the major advantages of this peptide-based system. Moreover, different ‘families’ of PA can be mixed to ‘dilute’ or ‘concentrate’ the density of the bioactive site on the surface of the fibres [218]. Firstly, introduced by Tirrell’s group in 1995 [219], PAs were extensively designed and used for TE by Stupp and co-workers, who applied PAs decorated with RGD, IKVAV, and GF-binding motifs for different applications in TE, including angiogenesis, neurogenesis, chondrogenesis, and enamel and bone regeneration [220,221,222,223].

## 6. Peptide-Based Hydrogels for IVD Repair

The good biocompatibility, tuneable physiochemical properties, and ease of injectability made SAPHs an attractive soft biomaterial for NP tissue engineering. Indeed, with the recent attention on NP regeneration as a therapeutic approach to treat early stage IVD degeneration, an increasing number of peptide materials have been explored as injectable NP hydrogel scaffolds. RADA16 systems, first introduced by Zhang et al. [184], represent the most used class of peptide hydrogels for NP regeneration. Li et al., for example, designed a new RADA16-based peptide (RKP) by attaching KPSS (KPSSAPTQLN), which is a short bioactive motif of bone morphogenetic protein-7 (BMP-7), to the C-terminal of a RADA16 for IVD TE applications [224]. KPSS was chosen since BMP-7 has been shown to stimulate the expression and deposition of PGs and collagen type II in vitro as well as induce an increase in disc height in degenerated discs in rabbits [225]. Dike’s group, for example, showed that transduced NP cells with human BMP-7, via adeno-associated virus-2 and injected into a canine model of disc degeneration, were able to effectively preserve the structural integrity, ECM, and biomechanical properties of the canine’s degenerated discs [226]. In a work by Li and colleagues, human NP-derived stem cells (NDSCs) were cultured in RKP and bare RADA16 peptide hydrogels in an apoptosis-inducing environment generated by the addition of TNF-α [224]. After 48 h, RKP effectively protected NDSCs from the pro-inflammatory TNF-α cytokine, promoting high viability and cell proliferation. Moreover, the hydrogel stimulated PG and collagen type II synthesis *in vitro*; it promoted upregulation of NP-marker genes, such as collagen type II, SOX9, and aggrecan and, more importantly, it restored disc height in rabbits affected by IVD degeneration [224]. Using the KPSS motif, Ruan’s groups showed that a mixture of RADA16 with KPSS-containing RADA16 in a 1:1 ratio promoted cell proliferation and chemotaxis of bone-marrow-derived MSCs as well as kept cell viability as a cell carrier up to 14 days once injected into an ex vivo cultured disc model [227]. In another two studies, Tao et al. took a further step in this direction with the use of BMP-mimetic sequences co-assembling RADA16 with RKP as well as RADA16 mixed with another two BMP-2 motifs, SNVILKKYRN and KAISVLYFDDS, and improved the therapeutic effect of RADA16, leading to higher gene and protein expression of NP cells [228].

Besides BMPs, Link N (peptide sequence: DHLSKNYTLDHDRAIH) represents another popular bioactive motif that has been used for NP TE applications. Link N has been shown to stabilise PGs in the ECM and to induce a GF-like effect on chondrocytes in vitro by stimulating the synthesis of PG and collagen type II [229]. Due to the presence of PG and collagen type II as major components of the NP’s ECM, it is not surprising that Link N has also been explored for NP studies. Wang et al., for example, conjugated Link N to RADA16 peptide hydrogel to form RLN and further co-assembled this with RADA16 to create a multicomponent peptide scaffold called LN-NS [230]. The authors showed that rabbit NP cells seeded on LN-NS migrated into the hydrogel and remained viable over time, while they did not with RADA16. Moreover, LN-NS hydrogels promoted a nearly two-fold increase in collagen type II and aggrecan gene expression compared with RADA16 only used as control [230]. Similarly, Ma et al. mixed RADA16 with Link N in a 1:1 ratio and showed that the new mixture promoted rabbit notochordal cell adhesion and the deposition of aggrecan and collagen type II after 14 days [231].

**Figure 9:**
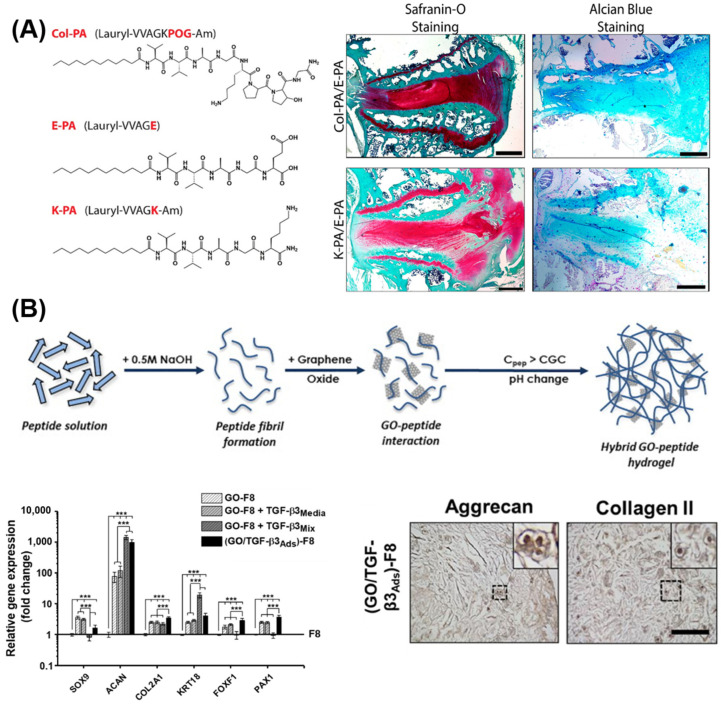
Representative studies employing peptide-based hydrogels for IVD-repair applications. (**A**) PA structures mimicking collagen fibres for IVD applications. MSCs cultured on PA nanofibres showed successful differentiation after 14 days and abundant GAG deposition (Safranin O and Alcian Blue stainings shown). (**B**) Self-assembly and formulation of GO-containing F8 hydrogels. TGF-β3-coated GO-F8 hydrogels showed increased gene and protein expression of NP cells after 3 weeks of 3D culture; *** *p*-value < 0.001. Reprinted and adapted with permission from Refs. [233,234]. Copyright 2022, Elsevier. Reprinted and adapted with permission from [236]. Copyright 2022, American Chemical Society.

Besides RADA-based systems, other β-sheet forming peptides have been explored for NP repair. Bian and colleagues, for example, formulated a physical mixture of KLD12 (dodecapeptide) and TGFβ1 for the culture of rabbit MSCs for disc regeneration, which showed good MSC viability and proliferation after 14 days of culture within the KLD12/TGFβ1 complex [190]. In this case, peptide hydrogels were able to induce MSCs differentiation into NP-like cells, and this was accompanied by an upregulation of aggrecan and collagen type II gene expression [190]. The same system alone was studied as an injectable scaffold for NP cells by Sun and co-workers. KLD12 self-assembled into long nanometric fibres (10–30 nm in diameter), which sustained rabbit NP cell viability for up to 14 days and increased the production of GAGs and collagen type II over time [232]. Shorter than 12 amino acids, we explored the use of the octapeptide FEFEFKFK for the 3D culture of bovine NP cells (BNPCs), as shown in Figure 9. A rheological study of FEFEFKFK revealed that the hydrogel stiffness could be tuned by increasing the peptide concentration (up to 35 mg mL^−1^) to mimic the native NP rheological properties [195]. BNPCs encapsulated in FEFEFKFK showed an upregulation of aggrecan, collagen type II, and novel NP-marker genes, such as FOXF1, KRT8, and KRT18, over 14 days of 3D cell culture [195]. Based on a similar sequence, recently, our group developed an injectable graphene oxide (GO) self-assembling peptide nanocomposite based on the β-sheet-forming peptide FEFKFEFK (F8) [233]. The octapeptide is a short octa-peptide that readily self-assembles into ∼3 nm-diameter β-sheet-rich fibres, which above a critical gelation concentration entangle/associate to form self-supporting, transparent, and injectable hydrogels. The incorporation of GO had a two-pronged objective. On the one side, GO flakes effectively increased the mechanical properties of peptide hydrogels to mimic the value of native NP tissue so that higher mechanical properties were achieved even with lower peptide concentrations (10–20 mg mL^−1^). On the other side, GO flakes could promote cell attachment and be decorated with relevant biomolecules to support cell viability and function. In a preliminary 3D cell culture study of 7 days, GO-F8 hydrogels were conducive to NP cell proliferation and preserved anabolic cellular activity, similar to those experienced by NP cells in vivo [233]. Due to the molecular design of F8, at a low pH, strong electrostatic interactions between positively charged F8 and negatively charged GO flakes drove the assembly of peptide nanofibres onto GO flakes, while at a physiological pH, hydrophobic interactions prevailed to create further hydrogel stiffening. These hydrogels were designed to shear-thin and recover their mechanical property instantaneously upon the removal of the injection’s shear forces and undergo further stiffening in contact with cell culture media [233]. In a subsequent study, GO flakes were decorated with TGF-β3, an important anabolic GF involved in disc formation and homeostasis [38]. Decorated GO flakes were incorporated in F8 hydrogels and compared with systems with TGF-β3 mixed within the hydrogel and TGF-β3 supplied exogenously. When TGF-β3 was anchored on GO’s surfaces, NP cells showed optimal matrix deposition after 21 days of culture, with a significant increase in gene expression (i.e., ACAN, COL2A1, FOXF1 and PAX1) and activation of SMAD signalling compared to peptide hydrogels with TGF-β3 exogenously added in the culture media or ‘free’ in the peptide hydrogels [234]. These two studies represent the first example of the potential advantages of merging peptide hydrogels with carbon-based nanofillers to achieve IVD repair and NP regeneration. Shorter than octapeptides, ultrasmall peptides (three to seven aminoacids) were also exploited by Hauser’s group and tested with porcine NP cells (pNPCs), rabbit erythrocytes, and human MSCs for IVD TE applications. The peptides in aqueous solution self-assembled via transitional α-helices that transformed into β-turn supramolecular structures. The formulated hydrogels resulted in NP tissue of two orders of magnitude stiffer than native porcine, but comparable with human NP. When incubated with the peptides, minimal hemolysis was observed, and when the peptides were neutralised to pH 7.4, pNPCs and hMSCs showed good viability and characteristic morphology [235].

Less explored for IVD regeneration, PA-based hydrogels were recently tested by Uysal et al. for NP-repair applications. In their study, Uysal et al. cultured rat MSCs in a PA, presenting the collagen-mimetic sequence Pro-Hyp-Gly (Figure 9) [236]. The hydrogels were reported in vitro to enhance MSC differentiation into NP-like cells, as observed by an increase in SOX9 over time. When injected into degenerated rabbit IVDs, the PA hydrogel induced more GAG and collagen deposition compared to saline solution and to the PA hydrogel lacking the POG motif [236]. The injected hydrogel provided a functional recovery of the rabbit IVDs, as shown by the degeneration index score obtained [236]. Among commercially available peptide hydrogels, Barreto-Henriksson et al. showed that PuraMatrix^®^ was able to support encapsulated MSCs’ GAG deposition over time as well as provide mechanical properties mimicking the native NP tissue [237]. As a potential tissue-engineering scaffold to reconstitute the NP in early degeneration, Moss et al. formulated a hydrogel composite based on an elastin-like polypeptide and thiol-modified hyaluronan [238]. The composite resulted in an injectable stiff hydrogel with time-dependant features desirable for injectable applications. Biologically, the hybrid hydrogel maintained high cell viability and maintenance of anabolic phenotype based on morphologic and immunohistochemical data [238]. Moreover, the hydrogels showed good biocompatibility in a preclinical rabbit annular puncture model [238]. More recently, Wilcox’s group formulated a peptide:GAG nanocomposite, where GAGs were vital components of the IVD’s ECM, regulating hydration and swelling pressure in vivo [239]. Peptide:GAG hybrid hydrogels were obtained by mixing four β-sheet tape-forming peptides (P_11_-4, P_11_-8, P_11_-9, and P_11_-12) with varying net charge and hydrogen bonding capacities with chondroitin sulphate, a proof-of-concept GAG present in the native NP. Specific peptides and specific peptide:GAG ratios were found to be key parameters to control gel rheological properties. In particular, peptide:GAG ratios of 1:2 and 1:10 matched human NP tissue properties and allowed injection in an ex vivo caudal disc model with minimal leakage from the injection site and fast in situ gelation [239]. Representative studies using self-assembling peptide hydrogels for NP tissue repair are collated in Table 3.

## 7. Summary and Conclusions

IVD degeneration is a degenerative disease that initiates within the NP, the gelatinous core of the disc, and ends up involving the entire vertebral segment. The disc loses its high level of hydration and, unable to withstand external stresses, it collapses while nerves and blood vessels start to infiltrate. Current treatments, based on either palliative care (e.g., steroids, NSAIDs) or artificial prostheses (e.g., discectomy), mainly target the symptoms but not the cause of the disease. This leads to poorly efficient treatments and co-morbidities. In the search for alternative therapy, peptide hydrogels could play a pivotal role in the treatment of NP defects, especially in the early stage of degeneration. Due to their inherent properties, peptide hydrogels can be mixed with cell suspensions (e.g., MSCs and/or NP cells), and quick and efficient gelation can be induced by mild external stimuli, such as contact with cell culture media. This approach allows the formulation of bioactive hydrogels with homogenous cell dispersion for downstream applications. Peptide hydrogels not only behave as protective carriers for encapsulated cells but, more importantly, they can be designed to serve as an instructive niche by depicting both mechanical (e.g., stiffness gradients) and biochemical cues (e.g., bioactive epitopes). The tunability of SAPHs allows them also to host nanofillers that can enhance or add functionality. In this context, the incorporation of graphene-based materials, for example, offers a valid example in which 2D nanomaterials could dramatically improve bulk mechanical properties, increase cell attachment, and act as a solid-phase presentation platform for relevant biomolecules, such as proteins and anabolic GFs [234,240,241]. Finally, the ability of SAPHs to be injected with excellent shear-thinning behaviour make them a suitable candidate for minimally invasive procedures and the spatial dispensing of hydrogels, for example, via minimally invasive punctures and bioprinting [199,242]. Taking these advantages together, we envisage that peptide hydrogels represent a promising tool to tackle IVD degeneration, especially when used as functional injectable fillers in the early stage of degeneration.

## Figures and Tables

**Table 1:** Morphological features and Pfirrmann grading system of IVD degeneration.

Grade	Structure Colour	NP/AF Distinction	Signal Intensity	IVD Height
I	Homogenous, bright white	Clear	Hyperintense	Normal
II	Inhomogeneous, opaque	Clear	Hyperintense	Normal
III	Inhomogeneous and grey	Unclear	Intermediate	Normal to slightly decreased
IV	Inhomogeneous, grey to black	Lost	Intermediate to Hypointense	Normal to moderately decreased
V	Inhomogeneous, black	Lost	Hypointense	Collapse of disc space

**Table 2:** Summary of advantages and disadvantages of natural vs. synthetic hydrogels.

Hydrogels	Relevant Examples	Main Advantages	Main Disadvantages
Natural	Hyaluronic Acid, alginate, chitosan, fibrin, gelatin, cellulose and gellan gum	Biodegradable Cell-binding sites Low immunogenicity Relatively cheap	Poor mechanical properties High degradation rates Batch-to-batch variability
Synthetic	poly(N-isopropylacrylamide), poly(ethylene glycol), poly(vinyl alcohol), poly(vinyl pyrrolidone)	Tuneable mechanical properties and shapes Tuneable degradation Ease of scalability	Lack of cell-recognition sites Toxic degradation by-products Toxic cross-linkers

**Table 3:** Studies reporting the use of peptide hydrogels as 3D scaffolds for IVD repair.

Hydrogel	Injectable?	Cell Type	Duration	Outcomes	Refs.
RKP (RADA16 functionalised with KPSS)	N/A	Human NP-derived stem cells	2 days	High cell proliferation and upregulation of the gene expression of collagen II, aggrecan, and Sox-9 compared to controls	[224]
RADA16:RADA-KPSS in 1:1 ratio	Yes	Human MSCs	14 days	Hydrogels promoted cell proliferation and chemotaxis as well as kept cell viability up to 14 days once injected into an ex vivo cultured disc mode	[227]
RAD-RKP (co-assembly of RADA16 with RKP)	Yes	Degenerated human NP cells	28 days	High cell proliferation and migration. Collagen type II, SOX9, and aggrecan were upregulated, while collagen I was downregulated compared to controls	[228]
LN-NS (co-assembly of link N-conjugated RADA16 with RADA16)	Yes	Rabbit NP cells	14 days	High viability and cell adhesion. Increased gene expression of PGs and collagen type II compared to controls	[230]
LN-NS (1:1 mixture of link N-conjugated RADA16 with RADA16)	Yes	Rabbit Notochordal Cells	14 days	High cell survival rate. Increased gene expression of aggrecan and collagen type II compared to controls	[231]
KLD12	Yes	Rabbit NP cells	14 days	Increased GAG and collagen type II production over time. NP cells preserved high viability and characteristic shape	[232]
KLD12/TGF-β1	N/A	Rabbit MSCs	14 days	High cell viability over time. Increased gene expression and protein deposition of aggrecan and collagen type II compared to controls	[190]
FEFEFKFK	Yes	Bovine NP Cells	14 days	Characteristic morphology and high viability over time. Upregulation of collagen type II, aggrecan, cytokeratin-8, cytokeratin-18, SOX9, and FOXF1, CA12	[195]
FEFKFEFK-GO	Yes	Bovine NP cells	7 days	Increased viability of NP cells and stable metabolic activity over time	[233]
FEFKFEFK-(GO + TGF-β3)	Yes	Bovine NP cells	21 days	TGF-β3-decorated GO flakes induced increased gene expression and matrix deposition over time compared to TGF-β3 ‘free’ in peptide hydrogels or added exogenously. SMAD signalling was preserved when TGF-β3 was anchored on GO flakes, inducing ECM production	[234]
Ac-ID_3_, Ac-LD_6_, Ac-AD_6_	N/A	Porcine NP cells	2 days	Porcine NP cells showed good viability when incubated with peptides	[235]
Col-PA/E-PA (PA hydrogel decorated with collagen-mimic POG motif)	Yes	Rat MSCs	14 days	*In vitro* differentiation into NP-like cells. Increased GAGs and collagen deposition compared to controls. Functional recovery of rabbit IVDs after injection *in vivo*	[236]
PuraMatrix^®^	Yes	Human MSCs	21 days	High deposition of GAGs accompanied by fast stress relaxation and mechanical properties mimicking native NP tissue	[237]
Elastin-like polypeptide/thiol-modified hyaluronan	Yes	Human IVD cells	21 days	High cell viability and maintenance of anabolic cell phenotype. Good biocompatibility in a preclinical rabbit annular puncture model	[238]
P_11_-4, P_11_-8, P_11_-9, P_11_-12/chondroitin sulphate	Yes	Ex vivo bovine caudal spine model	1 day	Hybrid hydrogels showed mechanical properties similar to the human NP. Hydrogels injected in a caudal spine model undergo gelation in situ with minimal leakage over time	[239]

## Data Availability

Not applicable.
